# The relationship between psychiatric symptoms and the use of levetiracetam in people with epilepsy

**DOI:** 10.1177/00912174231206056

**Published:** 2023-10-13

**Authors:** Omar Gammoh, Ahmed Al-Smadi, Mohammad Mansour, Wail Ennab, Suha AL Hababbeh, Ghaith Al-Taani, Mervat Alsous, Alaa AA Aljabali, Murtaza M Tambuwala

**Affiliations:** 1 Faculty of Pharmacy, Department of Clinical Pharmacy and Pharmacy Practice, 59179 Yarmouk University, Irbid, Jordan; 2 Faculty of Nursing, 61767 Al al-Bayt University, Mafraq, Jordan; 3 Department of Neurology, 275537 Al-Bashir Hospital, Amman, Jordan; 4 Faculty of Pharmacy, Department of Pharmaceutics and Pharmaceutical Technology, 59179 Yarmouk University, Irbid, Jordan; 5 Lincoln Medical School, Brayford Pool Campus, 4547 University of Lincoln, Lincoln, Lincolnshire, UK

**Keywords:** levetiracetam, epilepsy, psychiatric symptoms

## Abstract

**Background:** Mental health in people with epilepsy (PWE) is often overlooked, especially in developing countries.

**Purpose:** Consequently, the current work had two objectives: (1) to estimate the burden of depression, anxiety, insomnia, and stress, and (2) to examine the association of these psychiatric/psychological symptoms with levetiracetam and other relevant clinical factors in a cohort of Jordanian PWE.

**Research Design:** This is a cross-sectional study. The demographic and clinical data were recorded. Depression was measured by the Patient Health Questionnaire-9 (PHQ-9, Arabic-validated version) and anxiety by the General Anxiety Disorder-7 (GAD-7, Arabic-validated version). The insomnia severity index (ISI-A, Arabic version) was used to assess sleep quality, and the Perceived Stress Scale (PSS-A, Arabic version) was used to measure perceived stress.

**Study Sample:** Data were analyzed from 280 patients, of which 178 (63.6%) received levetiracetam as monotherapy or as adjuvant.

**Results:** Depression was reported in 150 (53.6%), anxiety in 110 (39.3%), insomnia in 131 (46.8%), and clinically significant stress in 211 (75.4%). At univariate analysis, levetiracetam was not associated with psychiatric symptoms. Multivariate logistic regression revealed that severe depressive symptoms were associated with family history (OR = 2.47, 95% CI = 1.42-4.33, *P* = .001) and seizure type (OR = 1.69, 95% CI = 1.01-2.80, *P* = .04), severe anxiety symptoms were associated with family history (OR = 1.90, 95% CI = 1.12-3.23, *P* = .01), severe insomnia was associated with seizure type (OR = 2.16, 95% CI = 1.33-3.5, *P* = .002) and severe stress was associated with marital status (OR = 2.37, 95% CI = 1.31-4.29, *P* = .004).

**Conclusions:** The high psychological burden of PWE is a challenging issue that requires attention and prompt action to control its risk factors. Levetiracetam was not associated with psychiatric symptoms in this study.

## Introduction

Epilepsy is a widespread neurological disorder with a prevalence reaching 1.1 persons per 1000 population in developing countries.^1^ Many people with epilepsy (PWE) suffer from psychiatric comorbidities that appear to be a complex interplay between psychosocial and neurobiological factors, behavioral, social aspects, cultural aspects and medications.^2-4^ Epilepsy itself is a stressor. Chronic stress associated with the disease is an important risk of developing other disorders, such as depression, anxiety, and insomnia.^5-7^

Depression and anxiety are the most frequent mental health disorders experienced by PWE.^8^ A study estimated that PWE had a higher rate of suicide by 50% as a result of severe depression.^9^ PWE feel anxious and concerned about their uncontrolled seizures, their onset and their helplessness during and after the seizure, and how this can affect their work, study, and daily activities.^10^

Although Antiseizure Medications (ASMs) are the mainstay for the treatment of epilepsy, their impact on mental health is gaining attention. It is as yet unclear whether the effects of ASMs on mood and behavior are an integral part of their mechanisms of action or a side effect. Some studies report a high prevalence of depression (8%–25%) and anxiety (17%–63%) symptoms for PWE using ASM.^11^ Other reports suggest that up to 20% of PWE receiving ASM experience depressive, psychotic, aggressive behaviors, and even suicide.^12,13^

Due to its pharmacologically favorable profile, including a relatively broad spectrum of efficacy against different types of seizures, the feasibility of rapid dose titration, the low risk of immune-mediated idiosyncratic adverse effects, the low teratogenic potential and the low potential to participate in drug-drug interactions, levetiracetam is an attractive first-line and adjunctive therapy in epilepsy.^14,15^

According to Chen et al. (2017),^12^ levetiracetam (LEV) had the greatest psychological and behavioral side effect rate (22.1%) compared to other ASMs. Moreover, levetiracetam users were sixfold more likely to develop depression. Another review^16^ reported a significant association between levetiracetam and depressive symptoms. Although the exact mechanism of the possible mood-related effects of levetiracetam is unclear, some studies indicated that levetiracetam acts on the release of presynaptic neurotransmitters by binding to synaptic vesicle protein 2A (SV2A), a glycoprotein that is part of the membrane of presynaptic neurotransmitter-containing vesicles in neurons and neuroendocrine cells.^17^ Moreover, levetiracetam could alter GABA, serotonergic, α2-adrenergic signaling paths and μ-opioid receptors.^18-20^

The study of the prevalence of different mental health symptoms and their corelates is important in the optimization of the health care service for PWE. In Jordan, very few studies have assessed mental health outcomes among PWE,^21^ and the possible impact of ASMs on the psychological symptoms has been never studied before in Jordanian PWE. Consequently, the current work had two objectives: (1) to estimate the burden of depression, anxiety, insomnia, and stress, and (2) to examine the association of these psychiatric/psychological symptoms with levetiracetam and other relevant clinical factors in a cohort of Jordanian PWE.

## Methods

### Study design and settings

Patients were recruited for a longitudinal study; however, the current work represents the analysis for their baseline status using a cross-sectional design. PWE attending the outpatient clinics at AL-Basheer Hospital in Amman during the period of June-September 2022 for follow-up visits were approached first using the convenient sampling method. Afterwards, inclusion criteria were applied. This research was approved by the Institutional Review Board (IRB, no.16/2022) and the IRB committee of AL-Basheer Hospital. All study methods were performed in accordance with the Declaration of Helsinki. Electronic written informed consent was obtained from all participants by pressing the “I accept to participate” button. Participants had the right to withdraw from the study at any time.

### Inclusion criteria

The inclusion criteria included patients diagnosed with epilepsy according to the American Academy of Neurology guidelines for at least one year, using the current ASM for at least one year, not diagnosed with any intellectual disability or with concomitant serious illnesses and currently not receiving any psychotropic medications.

### Study instrument

A self-administered structured online questionnaire was employed to cover the participants’ demographic and clinical data. The demographic data included sex, age, education level (high school education vs university education), marital status (single/not single) and employment status (yes/no). The clinical data included the presence or absence of family history of epilepsy, seizure type, and the severity of epilepsy. The severity of seizures was assessed using the Arabic version of the Chalfont scale.^22^ In order to evaluate the adherence to the ASMs, the Arabic validated version of the Morisky-4 scale was used.^23^ The scale is composed of 4 items which are behaviors associated with medication non-adherence, e.g., “do you forget to take your medication?” with simple scoring options as yes and no. The Arabic translation was demonstrated to be reliable with a Cronbach’s alpha score of .82. A score of 3 or more reflects adherence, while a score of 1 or 2 reflects nonadherence. Also, ASM self-satisfaction by the patients were assessed (a closed end question asking PWE if they are satisfied with their ASM with answers “Yes I am satisfied” and “No I am unsatisfied”).

### Exposure

Initially, the medical records of the patients were analyzed to determine the ASMs used by the patients. In order to ensure the accuracy of data collection, the ASM used was checked out by the patients from a checklist in the self-administered online questionnaire where the generic name and the picture of the medication pack were all demonstrated. The used and approved ASM list at the hospital included: levetiracetam, carbamazepine, lamotrigine, phenobarbital, phenytoin and valproic acid. The ASMs were then stratified into two groups: levetiracetam (monotherapy or adjuvant) and no levetiracetam.

## Outcome variables

### Depression

The Patient Health Questionnaire-9 (PHQ-9) Arabic validated version was used. The PHQ-9 is a short, self-administered scale based on the nine Diagnostic and Statistical Manual of Mental Disorders-IV criteria for diagnosing depression.^24^ The PHQ-9 has a sensitivity of 88% and specificity of 88% for severe depression, and was previously used in Arabic speaking MS patients, with a cut -off score of 15.^25^

### Anxiety

The General Anxiety Disorder-7 (GAD-7) is a short, self-administered scale with a cutoff point of 10 that has a sensitivity of 89% and specificity of 82% for diagnosing generalized anxiety disorder.^26^ The GAD-7 has previously been used in Arabic speaking MS patients with a cut-off score of 15.^25,27^

### Insomnia

The insomnia severity index Arabic version ISI-A was used to evaluate sleep quality. The ISI consists of 7 questions with Likert-type answers and a score range between 0 and 28 where a score above 15 indicates clinically significant insomnia. ISI is validated to be used in Arabic.^28,29^

### Perceived stress

The Arabic version of the Perceived Stress Scale (PSS-A) was used to screen perceived stress. The PSS was developed by Cohen and Williamson.^30^ It includes 14 items that are designed to measure individual stress for the last 30 days with a cutoff value of 14 reflecting clinically significant stress.

### Data analysis

Data were analyzed using SPSS software version 21. The distribution of the covariates between the study groups was analyzed using descriptive and frequency analysis and the Chi-Square test. We analyzed the distribution of potential confounding factors in the two exposure groups by frequency analyses and Chi Square test (Table 1). We also assessed the potential association between covariates and the outcome variables with univariate linear regression using a cut-off value of *P* < .10 to include covariates in the multivariate model. Subsequently, a multivariate logistic regression model was performed for each outcome variable including education, employment, family history of epilepsy, type of seizures, ASM self-satisfaction, and ASM adherence as adjustment variables. The confidence intervals were set at 95% and significance at *P*-value of .05.

**Table 1.** The study sample characteristics.

		No lev	Lev	
Factor	Category	n (%)	n (%)	*P*
Sex	Female	43 (30.3)	99 (69.7)	.02*
	Male	59 (42.8)	79 (57.2)	
Age	Less than 25	34 (30.6)	77 (69.4)	.06
	25 and above	68 (40.2)	101 (59.8)	
Marital status	Single	60 (37.3)	101 (62.7)	.42
	Married & others	42 (35.3)	77 (64.7)	
Education	High school	91 (37)	155 (63)	.37
	University level	11 (32.4)	23 (67.6)	
Employment	Unemployed	85 (34.8)	159 (65.2)	.10
	Employed	17 (47.2)	19 (52.8)	
Family history	no	55 (30.7)	124 (69.3)	.007*
	Yes	43 (46.7)	49 (53.3)	
Seizure type	Absence and other types	51 (35.7)	92 (64.3)	.44
	GTC	51 (37.2)	86 (62.8)	
Satisfaction with ASM	Poor	42 (40.8)	61 (59.2)	.15
	Moderate & high	60 (33.9)	117 (66.1)	
Adherence to ASM	Non-adhering	25 (46.3)	29 (53.7)	.06
	Adhering	77 (34.1)	149 (65.9)	

The distribution of the covariates between the study groups was analyzed using descriptive and frequency analysis and the Chi-Square test. “No Lev”: No levetiracetam included in the regimen, “LEV”: Levetiracetam included in the regimen. “GTC”: Generalized Tonic Clonic, ASM: Anti-seizure Medications,*Significance: *P* < .05.

## Results

### Study sample demographics, clinical details and psychiatric burden

Out of the 381 patients approached, 305 agreed to participate. 25 participants did not complete the study instrument and therefore data were analyzed from 280 patients. Almost half of the study sample were women 142 (50.7%), 25 years and older were 169 (60.4%), single were 161 (57.5%), those who had a family history of epilepsy were 179 (64%). The patients who reported receiving levetiracetam as monotherapy or adjuvant were 178 (63.6%). Regarding the severity of seizures, there was no difference between levetiracetam users (50.68 ± 25.01) and non-users of levetiracetam (49.12 ± 24.18) on the Chalfont scale, thus indicating PWE in our cohort had the same severity and therefore any differences in their mental health outcomes were unrelated to epilepsy severity. In regards to the psychiatric outcomes, significant depression was reported in 150 (53.6%), anxiety in 110 (39.3%), insomnia in 131 (46.8%) and clinically significant stress in 211 (75.4%). Table 1 shows the factors of the patients distributed across the ASMs.

### Association between ASMs and outcome variables

According to univariate logistic regression, there was no association between levetiracetam users and non-users with depression (uOR .96, 95% CI .59-1.57, *P* = .87), anxiety (uOR 1.13, 95% CI .69-1.86, *P* = .62), insomnia (uOR .84, 95% CI .52-1.38, *P* = .49) and perceived stress (uOR .73, 95%CI .42-1.27, *P* = .27). Please refer to Table 2.

**Table 2.** Association between levetiracetam and the psychiatric symptoms.

Depression			
	n (%) above cut-off	OR (95% CI)	*P*
Lev	96 (53.9)	.96 (.59-1.57)	.87
No lev	54 (52.9)		
Anxiety			
	n (%) above cut-off	OR (95% CI)	*P*
Lev	68 (38.2)	1.13 (.69-1.86)	.62
No lev	42 (41.2)		
Insomnia			
	n (%) above cut-off	OR (95% CI)	*P*
Lev	86 (48.3)	.84 (.52-1.38)	.49
No lev	45 (44.1)		
Perceived stress			
	N (%) above cut-off	OR (95% CI)	*P*
Lev	138 (77.5)	.73 (.42-1.27)	.27
No lev	73 (71.6)		

Univariate analysis between the Antiseizure medications groups and the psychiatric symptoms. Lev: levetiracetam included in the regimen, No Lev: NO levetiracetam is included in the regimen.

### Association between covariates and the outcome variables

According to the multivariate analysis, PWE were at higher risk for depression if they had a family history of epilepsy (aOR 2.47, 95% CI 1.42-4.33, *P* = .001) and if they had Generalized Tonic Clonic seizures (aOR 1.69, 95% CI 1.01-2.8, *P* = .045). However, higher education level and higher satisfaction with ASM were associated with lower risk of depression (aOR .35, 95% CI .15-.79, *P* = .01) and (aOR .52, 95% CI .31-.88, *P* = .015), respectively. PWE were at higher risk of anxiety if they had a family history of epilepsy (aOR 1.90, 95% CI 1.12-3.23, *P* = .017). High adherence was associated with a lower anxiety risk (aOR .31, 95% CI .16-.59, *P* = .001). Insomnia was associated with generalized tonic cleonic seizures (aOR 2.16, 95% CI 1.33-3.5, *P* = .002) and adherence to ASM (aOR .47, 95% CI .25-.87, *P* = .01). Perceived stress was associated with being married (aOR 2.37, 95% CI 1.31-4.29, *P* = .004). Please refer to Table 3.

**Table 3.** Association between covariates and the outcome variables using an initial univariate analysis followed by multivariate logistic regression models for each of the outcome variables (Depression, anxiety, insomnia, and perceived stress).

Depression				
Factor	Univariate		Multivariate	
	OR (95% CI)	*P*	OR (95% CI)	*P*
Education	.37 (.17-.79)	.01	.35 (.15-.79)	.01
Employment	.51 (.25-1.03)	.06		
Family history	2.56 (1.51-4.32)	.001	2.47 (1.42-4.33)	.001
Satisfaction with ASM	.54 (.33-.88)	.01	.52 (.31-.88)	.01
Adherence to ASM	.51 (.27-.95)	.03		
Seizure type	2.21 (1.37-3.57)	.001	1.69 (1.01-2.8)	.04
Anxiety				
	Univariate		Multivariate	
	OR (95% CI)	*P*	OR (95% CI)	*P*
Family history	1.99 (1.19-3.34)	.008	1.90 (1.12-3.23)	.017
Adherence to ASM	.30 (.16-.55)	.001	.31 (.16-.59)	.001
Seizure type	1.74 (1.07-2.82)	.02		
Insomnia				
	Univariate		Multivariate	
	OR (95% CI)	*P*	OR (95% CI)	*P*
Satisfaction with ASM	.62 (.38-1.0)	.05		
Seizure type	2.11 (1.31-3.41)	.02	2.16 (1.33-3.5)	.002
Adherence to ASM	.49 (.26-.89)	.02	.47 (.25-.87)	.01
Perceived stress				
	Univariate		Multivariate	
	OR (95% CI)	*P*	OR (95% CI)	*P*
Age	1.83 (1.05-3.17)	.03		
Marital status	2.37 (1.31-4.29)	.004	2.37 (1.31-4.29)	.004

## Discussion

“This study sought to examine the psychiatric burden in a convenience sample of PWE in Jordan. The purpose was to examine the association between levetiracetam and depression, anxiety, insomnia and perceived stress among Jordanian PWE.”

According to our findings, PWE reported a high psychiatric burden that is not associated with the use of levetiracetam. However, family history of epilepsy, education level, seizure type and adherence to ASMs were all associated with the psychiatric symptoms.

In the current study, depression, anxiety, and insomnia were reported in 40%-50% and perceived stress in about 75% of the study sample. In many settings, mental disorders of PWE are underdiagnosed and undertreated due to lack of awareness and factors such as lack of resources and patients such as epilepsy severity, unemployment, ignorance, and stigma.^21,31^ Compared to the literature, Jordanian PWE reported higher psychiatric symptoms compared to others. For example, depression and anxiety were tested in 17% among controlled PWE and more than 30% in refractory epilepsy.^32,33^ In the current study, insomnia was reported in 46% of PWE, which is in line with previous studies that used the same study design, sample size and insomnia measurement tool.^7^

In the current study, levetiracetam and other ASMs were not related to psychiatric outcomes, although the association between ASMs and psychiatric outcomes is evident in the literature. Levetiracetam is regarded in many studies as a risk factor for depression and other mood disorders in comparison to the other ASMs.^12,16^ Furthermore, a study that recruited more than a thousand PWE on levetiracetam reported depression in about 14% in two years.^34^ In addition, a recent study suggested that behavioral adverse effects improved after stopping levetiracetam discontinuation.^35^

On the other hand, two similar previous studies are in agreement with our findings. For example, levetiracetam users reported depression and nervousness similar to placebo (3.8% vs 2.1%) and (3.8% vs 1.8%) respectively.^36–38^ In addition, two other studies suggested a possible role for levetiracetam in alleviating anxiety and other disorders. For example, in a longitudinal study, levetiracetam use improved anxiety in refractory anxiety.^39,40^ The differences in the results from different studies may be due to differences in the study design, sample size, seizure severity, diseases duration, patient factors and the measurement tools of psychiatric symptoms, which are self-administered inventories.

In our study, PWE with a family history of epilepsy, ASM self-satisfaction and adherence were associated with poor psychiatric symptoms. The lack of control of epilepsy is associated with recurrent seizures, which can be related to social embarrassment and therefore to severe depression, anxiety, insomnia and stress. For example, in one study from Jordan, PWE with uncontrolled seizures reported higher odds ratios for depression and anxiety.^41^

Adherence to ASM is challenging and causes failure in ASM therapy.^42^ We found 84.8% of participants with moderate to high levels of adherence, while 15.2 exhibited low adherence to levetiracetam, respectively. Our results were similar to those reported by,^43^ which stated that 70% of epileptic patients had moderate to high levels of adherence to ASM. Depression was reported in more than half of the patients, which is consistent with the results of another study by.^44^ This needs further study and investigation. The results from the current study showed an association between educational accomplishment and depression. This was consistent with previous studies about quality of life, poverty and depression in patients who are taking ASMs, which showed an inverse relationship between depression and educational level.^45^ In Jordan, PWE reported lower years of education compared to their healthy peers. The educational achievement could reflect the intellectual capacity in school success, due to stigmatization. We suggest that PWE did not attend school frequently compared to their peers, which could explain depression severity.

### Study limitations

This study has some limitations. First, this is a cross-sectional study that does not confirm a conclusive cause-and-effect relationship. Second, some of the factors studied, like duration of epilepsy and family history of mental illness, are subject to recall bias. Third, the symptom similarity of epilepsy, depression and the adverse effects of ASMs overlap with symptoms of depression (such as fatigue and sleep disturbance) and may over represent the problem. Fourth, the exclusion criteria applied also limit the generalizability of the current findings to all PWE; for instance, our findings cannot be generalized for PWE with a diagnosis of less than one year. And finally, another limitation in the study is that some ASMs – such as carbamazepine and lamotrigine – could have psychotropic effects that may have interfered with the results^46^

## Conclusion

In conclusion, the present study did not demonstrate an association between levetiracetam and other ASMs and the high psychiatric burden in Jordanian PWE, more longitudinal studies are required to reveal any possible association.
